# Spatial and temporal dissociation of AQP4 and Kir4.1 expression during induction of refractive errors

**Published:** 2010-08-14

**Authors:** Melinda J. Goodyear, Sheila G. Crewther, Melanie J. Murphy, Loretta Giummarra, Agnes Hazi, Barbara M. Junghans, David P. Crewther

**Affiliations:** 1 School of Psychological Sciences, La Trobe University, Melbourne, Australia; 2 School of Optometry and Vision Sciences, University of New South Wales, Sydney, Australia; 3 Brain Sciences Institute, Swinburne University of Technology, Melbourne, Australia

## Abstract

**Purpose:**

Spatial co-localization of aquaporin water channels (AQP4) and inwardly rectifying potassium ion channels (Kir4.1) on the endfeet regions of glial cells has been suggested as the basis of functionally interrelated mechanisms of osmoregulation in brain edema. The aim of this study was to investigate the spatial and temporal changes in the expression of AQP4 and Kir4.1 channels in an avascular retina during the first week of the optical induction of refractive errors.

**Methods:**

Three-day-old hatchling chicks were randomly assigned to three groups and either did not wear lenses or were monocularly goggled with ±10D lenses for varying times up to 7 days before biometric assessment. Retinal tissue was prepared either for western blot analysis to show the presence of the AQP4 and Kir4.1 protein in the chick retina or for immunolocalization using AQP4 and Kir4.1 antibodies to determine the regional distribution and intensity of labeling during the induction of refractive errors.

**Results:**

As expected, ultrasonography demonstrated that all eyes showed rapid elongation post hatching. Negative lens-wearing eyes elongated faster than fellow eyes or normal non goggled eyes and became progressively more myopic with time post lensing. Positive lens-wearing eyes showed reduced ocular growth compared to normal controls and developed a hyperopic refraction. Quantitative immunohistochemistry revealed the upregulation of AQP4 channel expression on Müller cells in the retinal nerve fiber layer during the first 2 days of negative lens wear. Kir4.1 channel upregulation in the inner plexiform layer was only found on day 4 of positive lens wear during the development of refractive hyperopia.

**Conclusions:**

These results indicate that the expression of AQP4 and Kir4.1 channels on Müller cells is associated with the changes in ocular volume seen during the induction of refractive errors. However, the sites of greatest expression and the temporal pattern of the upregulation of AQP4 and Kir4.1 were dissimilar, indicating a dissociation of AQP4 and Kir4.1 function during refractive error development. Increased AQP4 expression in the nerve fiber layer is suggested to contribute to the rapid axial elongation and movement of fluid into the vitreous cavity in the presence of minus lenses; whereas, upregulation of Kir4.1 channels appears to play a role in limiting axial elongation in the presence of plus lenses.

## Introduction

The processes by which osmoregulation is maintained during rapid growth of organs such as the young eye are unknown. Indeed, fluid dynamics in the eye are not well understood [1–3], but it is generally accepted that osmoregulation of the retina is primarily controlled by solute-linked transport through the ion channels and transporter mechanisms of the retinal pigment epithelium (RPE) and the Müller glial cells that span the retina from the vitreal border to the sub-retinal space [4–9].

The importance of the Müller glial cells in retinal osmoregulation began to emerge after the discovery of specialized transmembrane water channels known as aquaporins (AQPs) [10]. AQP0, AQP1, AQP4, and AQP9 proteins have all been found in mammalian retina [11–14], but it is the AQP4 channel that has been localized to the Müller cell endfeet in rats [11,13] and chicks [15] and has been linked to the redistribution and absorption of ischemia-induced edema in the retina and brain [16–19].

AQP4 expression has also been reported to be co-localized with Kir4.1, the inwardly rectifying potassium channel, on the endfeet processes of astrocytes in the brain and retinal Müller cells [7,20]. Typically, these retinal regions of co-localized Kir4.1 and AQP4 channels act as potassium [K^+^] sinks for regulating high concentrations of [K^+^] in the extracellular space around active neurons [21,22]. This physical coincidence led to early suggestions of the coupling of water transport and K^+^ regulation by Müller cells [7, 20,23], though more recent studies have failed to demonstrate changes in Kir4.1 expression or K^+^ currents in AQP4 knockout mice [24].

The temporal and spatial relationship between AQP4 and Kir4.1 channel expression has not been explored thoroughly and never in a retina without intrinsic blood vessels, where the lack of blood vessels means K^+^ shunting is more tightly regulated by Müller cells. Indeed the absence of inner retinal blood vessels and astroglia allows a unique opportunity for the study of the expression of AQP4 and Kir4.1 on Müller cells. Thus it was chosen to investigate the spatial and temporal sequence of the expression of AQP4 and Kir4.1 channels during ocular growth in the avascular chick retina, where control of the axial length and volume have been linked to alterations in the rate of transretinal fluid transport [25,26].

Refractive compensation to minus lenses leads to abnormal increases in axial length and ocular volume and myopia; whereas, compensation to plus lenses leads to smaller than normal eyes with hyperopic refractive errors [27,28]. To date, there has been little consideration of the expression of proteins expected to be involved in the osmoregulatory mechanisms of fluid transfer that are required to achieve these rapid changes in ocular volume, apart from our recent reports demonstrating changes in the distribution of the ions across the retina [25,26] and an upregulation of AQP4 channel expression during the induction of form deprivation myopia [15]. This is the first study investigating the spatial and temporal changes in the expression of AQP4 and Kir4.1 channels in an avascular retina during the first week of the development of optically induced myopic and hyperopic refractive errors.

## Methods

### Animals and rearing

One hundred and two hatchling chicks (Leghorn/Australorp) were raised with unlimited food and water in a controlled environment on a 12 h:12 h light-dark cycle and with the temperature maintained at 30–31 °C from the day of hatching. All chicks were randomly assigned to one of three monocular goggle groups: No Lens (n=35), optical defocus of −10D goggles (n=33), or +10D goggles (n=34). Monocular defocusing goggles (±10D made with modified human polymethyl methacrylates (PMMA) contact lenses of 8.1 mm in diameter) were attached to Velcro^©^ and affixed to the periocular feathers for varying times from day 3: T=0 h (day 0; n=3), T=days 1–2 (n=37), days 3–4 (n=38), and days 5–7 (n=24). These particular time points were chosen to represent important times in the normal development of ocular growth and the early changes in growth seen in response to the introduction of ocular defocus [29]. Fellow eyes from experimental animals and both eyes from untreated age-matched No Lens animals acted as controls. Chick health and lens cleanliness were monitored twice daily. Retinal tissue was taken from four adult Hooded Wistar rats at 6 months of age. Rats were raised with unlimited food and water in a controlled environment on a 12 h:12 h light-dark cycle. They were killed by decapitation, and the tissue was then harvested for protein extraction within 1 min. All animals were raised and sacrificed in strict accordance with the La Trobe University Animal Ethics Committee guidelines. The regulations of the National Health and Medical Research Council of Australia, the ARVO Statement for the Use of Animals in Ophthalmic and Vision Research, and the United States NIH document “Guiding Principles in the Care and Use of Animals 1996” were followed.

### Biometric analysis

At the end of the period of defocus induction, biometrics were conducted using retinoscopy (Riester, Jungingen, Germany) to determine the refractive error and ultrasonography (Ophthasonic A-Scan III: 7 MHz probe, Teknar, Inc. St Louis, MO) to determine the axial length (anterior cornea to internal limiting membrane of the retina). All experimental procedures were performed under general anesthesia induced by an intra-muscular injection of a mixture of Ketamine (45 mg/kg) and Xylazine (4.5 mg/kg). Following anesthetic overdose and decapitation, the eyes from both the chicks and rats were enucleated and the anterior one-third of the eye was dissected away to reveal the eye cup. Posterior eyecups were immediately prepared for immunohistochemical labeling.

### Tissue preparation

Posterior eyecups were fixed in 4% paraformaldehyde for 30 min, washed three times in phosphate buffered saline (PBS; 0.01M phosphate buffer, 0.0027 potassium chloride and 0.137 M sodium chloride, pH7.4; Sigma-Aldich, St Louis, MO) for 5 min each, and incubated overnight at 4 °C with 30% sucrose for cryoprotection. Prior to sectioning, the eyes were embedded in O.C.T. compound (ProSciTech, Thuringowa, QLD, Australia) and frozen on dry ice. Embedded tissue was stored at −80 °C. Sections of the posterior eye cup (10 μm) were cut on a Leica CM 1850 cryostat (Leica Microsystems, Heidelberg, Germany) and thaw-mounted and dried on StarFrost positively charged slides (ProSciTech) before being stored at −20 °C.

### Immunohistochemistry

Immunolabeling of AQP-4 and Kir4.1 was conducted using immunohistochemistry as previously described [15]. Briefly, following rehydration with PBS, sections were incubated for 1 h in a 3% goat serum blocking solution with Triton X-100, followed by primary antibody application overnight at 4 °C with either rabbit anti-rat AQP4 antibody (1:500; Chemicon, Temecula, CA) or rabbit anti-rat Kir4.1 antibody (1:333; Chemicon) and rabbit anti-Glial Fibrillary Acidic Protein (GFAP; 1:100 or 1:500; Sigma, Carlsbad, CA), a reactive marker for Müller cells. Sections were then incubated for 1 h at room temperature with Alexa 596-conjugated goat antirabbit IgG (Molecular Probes, Invitrogen, Eugene, OR) diluted to 1:400. Negative control sections were processed with the primary antibody omitted from one section per slide. All sections were coverslipped with gel mount mounting medium (ProSciTech). Labeled sections were observed under a Leica TCS-SP2 confocal laser scanning microscope (Leica Microsystems). Labeling at the posterior pole was examined for both regional distribution and change in intensity. Sections were viewed at 40× magnification and images captured with the Leica software set at 6× full frame averaging. Images were obtained from pre-determined photomultiplier settings (700 V and 750 V) and constant laser intensity for comparisons of the intensity of labeling between the optically defocused eyes and control eyes.

### Image analysis

Mean luminance values for labeling were determined from brightness histograms, as it is generally accepted that pixel intensity is proportional to chromagen concentration under particular defined conditions [30–32]. In all images, the inner retina was labeled brightest; hence, labeling intensity (luminance) was analyzed for the nerve fiber layer (NFL) and the inner plexiform layer (IPL). For both experimental and control groups, mean luminance values were obtained from the luminance histogram determined using the NIH Image (National Institute of Health, Bethesda, MD) to select the area for analysis across the breadth of one field of view for the NFL and IPL. The fellow control eye was chosen as the point of comparison in all analyses to control for between-animal differences in eye size.

### Western blot analysis

To obtain material for western blot analysis, retinal tissue (retina/RPE/choroid) was obtained from three normal white leghorn chicks and from three adult Hooded Wistar rats for comparisons between species. The extraction of proteins was conducted by homogenization of tissue in ice-cold lysis buffer containing 20 mM Tris-HCl (pH 7.5), 150 mM NaCl, 1 mM EDTA, 0.5% (V/V) Triton X-100, 0.1% (W/V) sodium dodecyl sulfate (SDS), and protease inhibitors (Complete Protease Inhibitor Cocktail; Roche Molecular Biochemicals, Indianapolis, IN). Supernatant was collected and stored at −20 °C. Protein content was determined using a Pierce Protein Assay Kit (Pierce, Waltham, MA). For SDS–PAGE, protein samples (120 µg/lane) were diluted in Laemli buffer (Sigma-Aldrich, Bio-Rad Laboratories, Gladesville, NSW, Australia) and resolved on a ready-made 12% TRIS-HCl gel (Biorad Laboratories). Following electrophoresis, immunoblotting was performed by electrotransferring the protein to a Nitrocellulose membrane (Millipore, Billerica, MA) in transfer buffer (25 mM Tris, 192 mM glycine, 20% methanol [v/v], pH 8.3). The membrane with blotted proteins was blocked for 1 h at room temperature with Tris buffered saline (TBS) containing 5% (w/v) skim milk powder, 1% (w/v) BSA (BSA), and 0.1% TWEEN 20, followed by incubation overnight at 4 °C with polyclonal rabbit antirat AQP4 (diluted 1:1,000; Chemicon International). After four washes with TBS containing 0.05% TWEEN 20, the membrane was incubated with a 1:2,000 dilution goat anti-rabbit IgG (whole molecule) horse radish peroxide (HRP) conjugated antibody (Sigma-Aldrich). Peroxidase activity was visualized using Lumiglo chemiluminescence reagents (Roche) processed on autoradiographic film. Blots were re-probed with β-actin antibody (1:10,000; mouse monoclonal antibody; Sigma) to verify equal protein loading.

### Statistical analysis

ANOVA (SPSS) was performed between the optically defocused eye and the fellow control eye to determine differences in labeling intensity according to the degree of lens defocus applied and the length of defocus time. Post hoc analyses were undertaken using either Student–Newman–Keuls or Games-Howell tests where appropriate. Quantitative data are presented as means±SEM.

## Results

### Axial length and refraction changes

**Figure 1:**
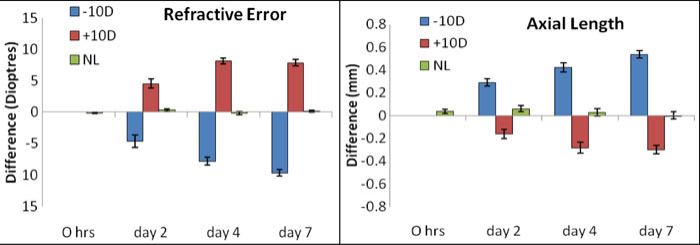
Mean ocular difference (Experimental – Fellow eye) of refractive error and axial length properties of chick eyes following various periods of lens wear [minus lenses (−10D), plus lenses (+10D), or No Lens (NL) groups]. Note that substantial axial length changes and refractive error developed within the first two days of lens wear. Minus lens wearing eyes showed more rapid changes in axial length in comparison to plus lens wearing eyes. Note also that the refractive compensation to plus lenses was maximal after 4 days; whereas, minus lens-wearing eyes continued to become more myopic over the 7 days examined.

As evident from Table 1 and Figure 1, all chick eyes examined continued to grow in the first week of life, though the rate of growth varied with the sign of the applied defocus. Relative axial elongation for minus lenses was greater than the relative shortening observed with plus lenses (see Figure 1 and Table 1). Concurrent with the axial length changes, lens-wearing eyes showed refractive compensation to the sign of the applied defocus (see Figure 1 and Table 1). No change in refraction was seen between right and left eyes in the No Lens control group.

**Table 1:** Mean (±SE) Ocular Axial Length and Refractive Error after varying periods of visual deprivation in the chick.

**Time (days)**	**Number of eyes**	**Eye**	**Axial length (mm)**	**Mean refraction (D)**
0	3	No Lens	8.30 (±0.05)	−0.25 (±0.52)
	3	No Lens fellow	8.27 (±0.04)	−0.08 (±0.58)
Day 2	11	Minus lens	8.88 (±0.07)	−4.00 (±1.10)
	11	Minus lens fellow	8.59 (±0.07)	0.66 (±0.37)
	15	Plus lens	8.44 (±0.07)	5.72 (±0.62)
	15	Plus lens fellow	8.60 (±0.07)	1.20 (±0.31)
	11	No Lens	8.64 (±0.08)	1.11 (±0.51)
	11	No Lens fellow	8.58 (±0.08)	0.77 (±0.44)
Day 4	14	Minus lens	9.34 (±0.05)	−7.32 (±0.65)
	14	Minus lens fellow	8.92 (±0.03)	0.48 (±0.35)
	12	Plus lens	8.45 (±0.06)	8.48 (±0.57)
	12	Plus lens fellow	8.73 (±0.07)	0.38 (±0.42)
	12	No Lens	8.79 (±0.05)	−0.04 (±0.45)
	12	No Lens fellow	8.78 (±0.06)	0.15 (±0.35)
Day 7	8	Minus lens	9.29 (±0.03)	−9.31 (±0.41)
	8	Minus lens fellow	8.75 (±0.03)	0.34 (±0.30)
	7	Plus lens	8.52 (±0.06)	8.32 (±0.60)
	7	Plus lens fellow	8.82 (±0.04)	0.46 (±0.38)
	9	No Lens	8.91 (±0.04)	0.31 (±0.34)
	9	No Lens fellow	8.90 (±0.04)	0.17 (±0.29)

Optically defocused eyes showed significant sign-dependent differences in eye size (*F*(2,36)=43.49, p<0.0001) and refractive error development (*F*(2,36)=40.74, p<0.0001) as early as day 2 of lens wear and continuing for the week of refractive error induction.

### AQP4 and Kir4.1 protein expression in the retina

**Figure 2:**
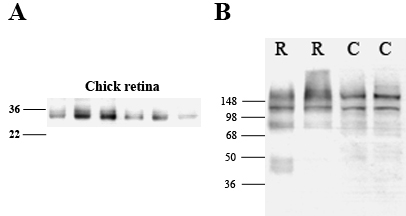
Western blot analyses of retinal tissue demonstrating antibody specificity to aquaporin water channels (AQP4; A) and Kir4.1 (B) in rat (R) and chick (C) retina. **A**: For AQP4, the appearance of bands between 30 and 32 kDa represents the estimated size of AQP4 in the chick eye [15]. **B**: For Kir4.1, bands were present in both normal rat and chick at approximately 85 kDa, representing the dimeric form of Kir4.1. Additional bands were also seen at approximately 200 kDa, which were presumed to represent the tetrameric form of Kir4.1 [24,33]. The lanes correspond to retinal tissue from normal chicks.

Immunoblot analysis of normal rat and chick retinal tissue demonstrated expression of the proteins AQP4 and Kir4.1 in both species. Bands representing a molecular weight of 30–32 kDa, which is the estimated size of AQP4, were present in the chicks and have previously been shown in the rat retina [15]. Bands immunopositive for Kir4.1 antibodies at a molecular weight of approximately 85 kDa were presumed to represent the dimeric form of Kir4.1 [24]. Additional bands were seen at approximately 200 kDa in both the rat and chick (see Figure 2) and were presumed to represent the tetrameric form of Kir4.1 [33].

### Immunolocalization of AQP4 and Kir4.1 channel expression in the normal chick retina

**Figure 3:**
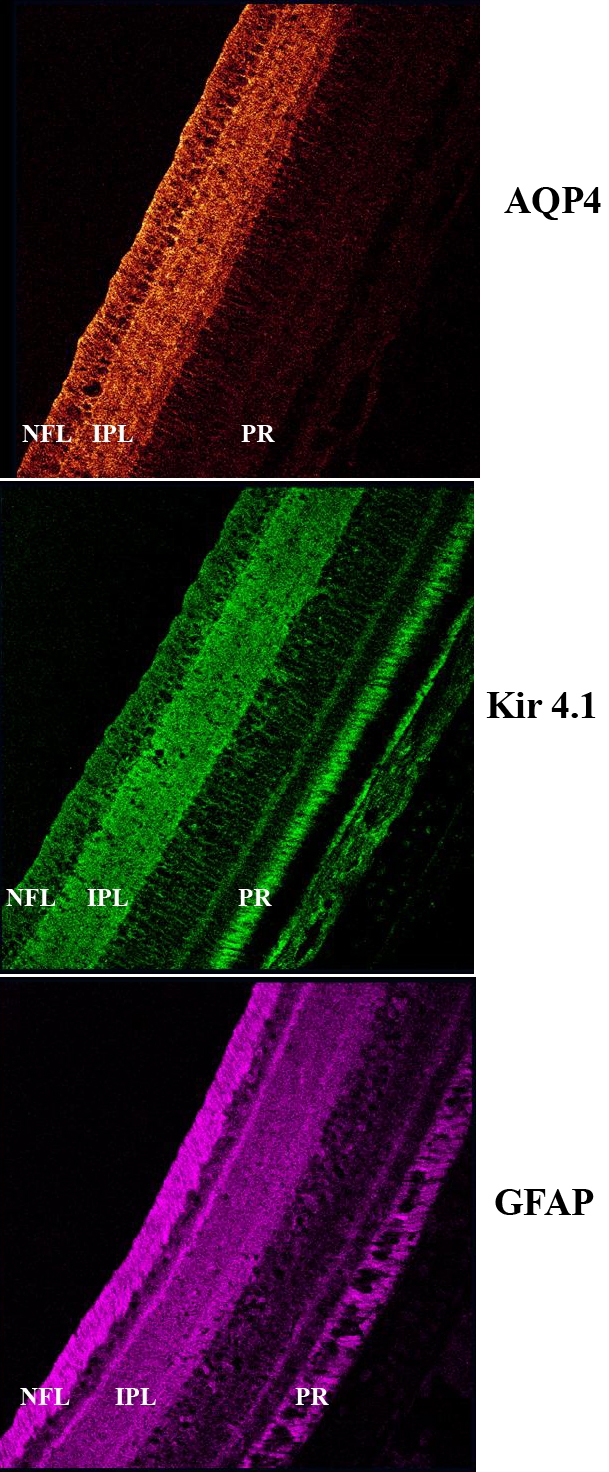
Immunolocalization of aquaporin water channel AQP4, inward rectifying potassium Kir4.1 channel, and glial fibrillary acidic protein (GFAP) expression in the chick retina. Staining of AQP4 (red) and Kir4.1 (green) is similar to that of GFAP (purple), a known marker of Muller cell processes. The layers of the retina are indicated as NFL for nerve fiber layer, IPL for inner plexiform layer and PR for photoreceptor layer.

**Figure 4:**
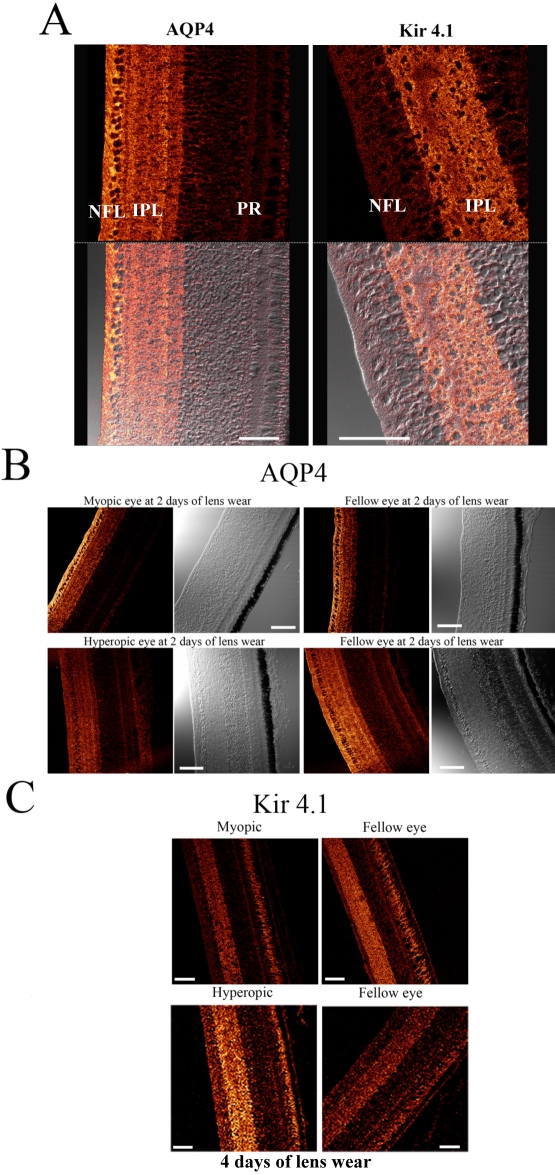
**A**: Magnified images of the immunolocalization of anti-aquaporin water channels (AQP4) and anti-Kir4.1 antibodies in the inner chick retina, demonstrating differences in the spatial distribution of AQP4 and Kir4.1 (Upper panels show confocal images illustrating the immunodistribution of AQP4 and Kir4.1 channel expression in normal chick retina. Lower panels show the associated differential interference contrast images). AQP4 expression was prominent in the nerve fiber layer (NFL), ganglion cell layer (GCL), and inner plexiform layer (IPL), whereas, Kir4.1 expression was more prominent in the IPL with some diffuse staining evident in the NFL/GCL and branching processes extending out into the inner nuclear layer (INL). The scale bar represents 50 µm. **B**: AQP4 expression following 2 days of lens wear. Upper images- Confocal microscopy showing AQP4 labeling and the accompanying differential interference contrast images from the myopic (left) and fellow eye (right) 2 days post lensing. Lower images - Similar confocal and interference contrast images of AQP4 labeling in hyperopic and fellow eyes. The scale bar represents 50 µm. **C**: Kir4.1 immunoreactivity after 4 days of lens wear. Upper panels show Kir4.1 immunopositivity in the myopic and fellow eye. Lower panels show Kir4.1 immunopositivity in the hyperopic eye and fellow eye. Note the appearance of strong labeling in the IPL of the hyperopic eye compared to the fellow eye below and lesser staining of Kir4.1 in the myopic eye compared to the fellow eye. The scale bar represents 50 µm.

In normal eyes, the immunopositive labeling of AQP4 and Kir4.1 was consistent with the GFAP staining of the filamentous processes of the Müller cells (see Figure 3). Staining for AQP4 was found on tightly wrapped bundles around individual ganglion cell soma and interweaving branching processes (see Figure 3 and Figure 4A), matching the fine filamentous processes of avian Müller cells as previously described [34]. The distribution of the immunolabeling of Kir4.1 also corresponds with avian filamentous processes extending from bifurcated processes, appearing to branch out and divide into numerous processes at the margin of the IPL (Figure 3 and Figure 4A) [34].

The pattern of distribution of AQP4 expression across the retina was similar in all groups examined. The most intense AQP4 immunoreactivity was seen in the inner retina along near the vitreo-retinal interface including NFL, ganglion cell layer (GCL), and IPL (see Figure 3 and Figure 4A). Within the IPL, AQP4 staining was apparent in both sublaminae A and B [34–36]. AQP4 labeling was minimal within the outer plexiform layer, outer nuclear layer, inner segments, outer limiting membrane, and outer segments of the photoreceptor layers. Diffuse background fluorescence was seen around the photoreceptors. A similar intensity of non-specific labeling in the outer retina was also seen in the negative control slides with the primary antibody omitted.

Kir4.1 immunoreactivity was seen in the inner retinal layers, with prominent staining occurring within the IPL and less intense staining evident in the GCL, NFL, and vitreo-retinal interface (see Figure 3 and Figure 4A). Consistent with AQP4 staining, Kir4.1 staining was evident within the sublaminae A and B of the IPL, and appeared similar to AQP4 labeling. Diffuse labeling of Kir4.1 was also present in the region of the outer limiting membrane and around photoreceptors. Less diffuse background fluorescence was also seen in negative control slides where the primary antibody was omitted.

While the labeling of AQP4 and Kir4.1 was predominantly found in similar regions of the retina, the localization of the greatest intensity of AQP4 and Kir4.1 labeling differed within the chick retina. The greatest intensity of AQP4 staining was seen in the NFL; however, the greatest intensity of Kir4.1 immunoreactivity was always present in the IPL (see Figure 3 and Figure 4A).

### Pattern of AQP4 immunoreactivity during the induction of refractive error by optical defocus

**Figure 5:**
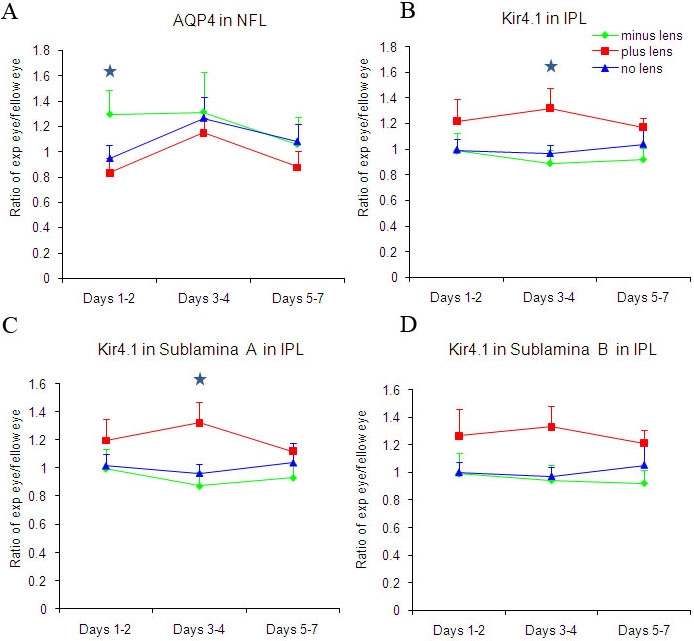
Comparison of the spatial and temporal distribution of AQP4 and Kir4.1 channel expression during the induction of refractive compensation to optical defocus (using pixel intensity analysis of stained sections). Blue stars indicate significant differences between the lens groups and/or control (No Lens) group (*p<0.05). Figures show the ratio mean luminance levels from the right eye (experimental eye) compared to the fellow left eye. **A** is the mean ratio luminance levels for AQP4 in the nerve fiber layer (NFL), **B** is the mean ratio luminance levels for Kir4.1 in the inner plexiform layer (IPL), **C** is the mean ratio luminance levels for Kir4.1 in sublamina **A** in the IPL, and **D** is the mean ratio luminance levels for Kir4.1 in sublamina **B** in the IPL.

A similar regional distribution of AQP4 labeling was found between myopic, hyperopic, and No Lens eyes, though there were differences in the intensity of labeling between groups. Early in the development of myopia (within the first 2 days of minus lens wear) and during the greatest increase in axial size and refractive error (see Figure 1), a significantly greater expression of AQP4 was seen in myopic eyes (as a ratio of experimental to fellow eyes) compared to hyperopic eyes in the NFL (*t*(21)=2.142, p*<*0.05; Figure 4B and Figure 5A). Positive immunoexpression in both hyperopic eyes and No Lens controls did not significantly differ in either the NFL or IPL (Figure 4B and Figure 5A). This difference in AQP4 expression in minus defocused eyes only existed early in development, and by day 4, no differences were seen in AQP4 expression. This lack of difference coincides with the general decrease in the rate of eye growth that begins after the compensatory myopic refractive error has been well established, as shown in Figure 1. No significant differences were seen in the level of AQP4 expression in the IPL, suggesting that AQP4 upregulation only occurs at the endfeet of Müller cells at the vitreal border in growing eyes.

### Kir4.1 immunoreactivity during the induction of refractive error by optical defocus

Kir4.1 labeling also showed similar regional distributions in all eyes irrespective of the induced refractive error. Changes in the intensity of expression of Kir4.1 protein in the experimental eye relative to the fellow non-lensed eye only became statistically significant after 4 days of lens wear. After 4 days of lens wear a main effect for Kir4.1 labeling in the IPL was seen [*F*(2, 25)=3.813, p<0.05]. This effect was also found to be significant for the Kir4.1 staining of sublamina A of the IPL [*F*(2, 25)=4.365, p<0.05], but not for that of sublamina B, suggesting that changes in the expression of Kir4.1 in the experimental eye relative to the fellow control eye occur within this sublamina of the IPL.

Post hoc analyses showed that significantly higher Kir4.1 expression was seen in the IPL and particularly in sublamina A in hyperopic eyes compared to myopic eyes (*t*(18)=-2.305, p*<*0.05; Figure 4C and Figure 5C,D). The IPL showed increased Kir4.1 expression in hyperopic eyes compared to myopic and No Lens eyes; however, sublamina A showed the greatest significant change across groups (Figure 5B-D). Myopic eyes and No Lens control eyes did not differ significantly in terms of Kir4.1 labeling luminosity (Figure 4C and Figure 5B).

## Discussion

Here, we report a spatial and temporal dissociation in the expression of AQP4 and Kir4.1 channels during the early changes in ocular growth rates accompanying the optical induction of refractive errors. This is the first time AQP4 and Kir4.1 channel expression has been examined concurrently in an in vivo model during the development of optical defocus-induced changes in the rate and volume of eye growth. It is readily apparent that an upregulation of Kir4.1 was not associated with a concomitant increase in AQP4 channel expression; rather, we have demonstrated a dissociation of the expression of AQP4 and Kir4.1 channels on Müller cells in the avascular retina.

Abnormal axial elongation induced by negative optical defocus was associated with an early upregulation of AQP4 channel expression in the NFL but not in the IPL; whereas, restriction in axial elongation induced by plus lens defocus was associated with an upregulation of Kir4.1 channels in the IPL but not in the NFL later in the induction of hyperopia. Furthermore, we have shown an association between the expected rapid increase in ocular volume and myopic refraction and AQP4 expression in the NFL, but no significant changes in Kir4.1 expression were found compared to No Lens eyes. However, during the induction of hyperopic refractions and the associated restricted ocular growth, Kir4.1 channel expression was only found to increase at days 3–4 even though AQP4 expression did not significantly differ from that measured in the No Lens eyes in any layer. As suggested earlier, this dissociation was not entirely unexpected because such a dissociation has been reported previously [24]. In a recent in vitro patch clamp study of isolated Müller cells, Ruiz and colleagues report little effect on Kir4.1 channel activity and no changes in Kir4.1 expression or K^+^ currents in AQP4^−/−^ mice [24].

In the present study of the avascular chick retina, Kir4.1 expression was predominantly found on Müller cell processes extending throughout the inner retina, from the vitreo-retinal border and at the outer limiting membrane. Enhanced expression localized to sublaminae within the IPL has been assumed to be associated with Kir channels on the horizontal processes of the Müller cell [34]. The lamination and division of the numerous fine filamentous processes in the IPL of the avian retina are believed to represent a specific adaptation to the high need for the spatial buffering of K^+^ ions following neuronal activity in the extracellular space of the inner retina in the absence of retinal blood vessels and associated astroglia [34]. Thus, in the present study, the enhanced expression of Kir4.1 in the IPL in hyperopic eyes may suggest that Kir4.1 function in the chick exhibits properties relating to K^+^ influx, as opposed to the efflux properties suggested to be characteristic of Kir4.1 channels located at the vitreo-vascular interface in vascularized mammalian retinas [37]. Indeed, Kir4.1 is known to facilitate both inward and outward K^+^ currents [38]. The increase in Kir4.1 expression found in the chick IPL during hyperopia could be in response to increased K^+^ release in the extracellular space surrounding synapses following neuropil activation in a retina lacking inner retinal capillaries. Further, the enhanced Kir4.1 expression was found particularly in sublamina A of the IPL, which could reflect an increase in K^+^ release in the extracellular space surrounding OFF center bipolar ganglion cell synapses following activation of the OFF pathway under the influence of positive lenses [36]. Interestingly, our previous biometric studies have also demonstrated that inhibition of the OFF response by pharmacological suppression results in the inhibition of hyperopic development to plus lenses [39,40]. Thus, enhanced Kir4.1 expression in the inner retina appears to be functionally linked to reduced axial elongation and growth and refractive compensation to plus lens defocus. By comparison, no significant difference in Kir4.1 expression was observed in normally growing No Lens eyes or the faster growing minus lens-wearing myopic eyes.

As expected from our earlier studies that demonstrated upregulated AQP4 channel expression during form-deprivation myopia (FDM) [15], the elevation of AQP4 at the vitreo-retinal border during minus lens-induced myopic development was also seen here. These findings support our earlier suggestion that myopic development is associated with the upregulation of water channels during the time of greatest axial growth changes [15,41]. Such an increase in AQP4 channels at the vitreo-retinal border would support enhanced water movement from the inner retina to the vitreous cavity via the Müller cells [11]. In addition, the timing of this upregulation corresponds with the time of greatest change in myopic refractive development.

As AQPs are known to facilitate transcellular water movement in response to osmolarity changes [42,43], the AQP4 upregulation observed during the induction of myopia in the present study is consistent with previously described changes to ion and water distribution patterns in FDM [25,26]. Recovery from FDM has been shown to be accompanied by rapid dissipation of the abnormally high levels of potassium ions in the sub-retinal space and outer retina and more gradual dissipation of elevated sodium and chloride ion levels in the inner and outer retina and choroid [26]. Ultrastructural studies also show the expansion of the choroidal blood vessels and lymphatics [25,26] during refractive recovery and return of ionic homeostasis. The results of the present study showing a change in the expression of both AQP4 and Kir4.1 channels during imposed optical defocus provides further support for the involvement of osmoregulatory mechanisms in ocular growth. To date, the osmoregulatory mechanisms associated with the induction of hyperopia have received little attention; in fact, this study is the first to examine the changes in expression of some osmoregulatory mechanisms during the induction of hyperopic refractive errors. Further studies investigating the change in transretinal fluid movements and the associated osmoregulatory mechanisms underlying hyperopic development are necessary to elucidate this area more completely.

In summary, we have reported significant changes in the expression of molecular mechanisms associated with retinal osmoregulation and water movement, i.e., AQP4 and Kir4.1 expression within the inner retina in response to imposed defocus and rapid changes in ocular volume. The results demonstrate that the upregulation of AQP4 channel expression in the NFL is likely to be the conduit for water movement during the most rapid expansion of the vitreous chamber volume during early development of myopia. Furthermore, the later increase in the expression of Kir4.1 channels in the IPL of the retina, particularly in sublamina A, during the restriction of ocular growth is likely to be in response to the need for potassium movement out of the extracellular space and around the OFF pathway neurons as a consequence of refractive compensation to plus lens defocus.
